# Members' Conference

**Published:** 1919-10-11

**Authors:** 


### THE INCORPORATED SOCIETY OF TRAINED MASSEUSES.

### Members' Conference.

Despite many obstacles which threatened postponement, the Conference of Members of the Incorporated Society of Trained Masseuses was held on Thursday, Friday, and Saturday, October 2, 3, and 4. The Committee were fully justified in their decision against its cancellation by the numerous and appreciative audiences at each sitting. The Conference was opened on Thursday morning at the Royal Society of Arts, John Street, Adelphi, and Miss Humble, in a few well-chosen words, welcomed all who had made the successful effort and were present —several members having arrived who had ventured the journey from Leeds. An interesting programme had been arranged, including lectures and demonstrations; but, owing to the unforeseen circumstances, this had to be slightly altered.

The speaker on Thursday morning was Captain Wood-Jones, R.A.M.C., whose subject was entitled "How we know ourselves." In the discourse the speaker traced a structural edifice of how information concerning their own being, and that of the outside world is conveyed to animals and to the human mind by the use of the senses and the mental impressions, illustrating the term "Mental Pictures" or pictured movements, and introducing the newer term "Action-patterns." The lecture was listened to with great interest and pleasure, and, contrary to the rules of the Society, a vote of thanks was accorded to Captain Wood Jones.

The meeting met later at 2.30 p.m. at St. Bartholomew's Hospital, where Major Elmslie, R.A.M.C., gave as his subject "Deformities of the Spine." After the lecture the members were shown the electrical, x-ray and massage departments, also the museum. In each section a demonstration was given of the use of the many appliances by the staff, who were unstinting in imparting information of every kind. The hospitality of St. Bartholomew's was further extended by the kindness of the matron, who entertained the whole party to tea in the Great Hall. The meeting was resumed at six o'clock in the Armitage Hall, Great Portland Street, the lecturer being Captain Humphris, R.A.M.C., whose subject, "Diathermy, with notes on the melted paraffin-wax bath and the tungsten arc light," was intently followed.

The meetings on Friday were all held at the Royal Society of Arts, John Street, Adelphi, and the attendance was much larger than on the previous day. To an appreciative audience, Dr. Justina Wilson spoke on the "Diseases of the Respiratory System," illustrating her subject by screen pictures, and most ably discussing the necessity and the benefits of deep-breathing exercises. In the afternoon the subject was the "Treatment of Fractures by Massage from the civilian point of view," given by Dr. Agnes Keene; and at the evening session a discourse on "Stiffness of Joints," by Colonel A. H. Tubby, C.B., C.M.G.

The Conference was brought to its conclusion on Saturday morning by a visit to St. Thomas's Hospital, an occasion which proved as instructive as it was pleasurable. Miss Randell, sister-in-charge of the massage and electrical departments, had travelled from the West of England in all the adverse circumstances of disorganised railway conditions, and at very short notice had most kindly consented to give the members of the I.S.T.M. a demonstration of the methods used in the re-education of the muscles and their adaptation to splints and artificial limbs after injury or amputation. The company were divided into four groups, each group piloted by a guide through all the wonderful departments devoted to diathermy and massage, orthopædic work, the plaster splint rooms, and the gymnasium. In the latter officer patients demonstrated the instruction essential to the proper use of the artificial limb, the correct manipulation in walking, climbing steps, and the estimation of distance.

There can be no doubt that the Conference in the year 1919 was most successful; the attendance was good; the audiences were animated and appreciative of the subjects brought before them; the lecturers at all times interesting. The Conference closed with a call to the members for more individual effort in the reconstruction of the I.S.T.M., for loyalty, and *esprit de corps*, for the sake of good work and the ultimate attainment of a National School of Massage.

